# Ratio-based estimators for a change point in persistence

**DOI:** 10.1016/j.jeconom.2012.05.024

**Published:** 2012-11

**Authors:** Andreea G. Halunga, Denise R. Osborn

**Affiliations:** a Department of Economics, University of Exeter Business School, Rennes Drive, Exeter EX4 4PU, United Kingdom; b Economics, School of Social Sciences, University of Manchester, Manchester M13 9PL, United Kingdom

**Keywords:** Persistence change, Order of integration, Structural breaks

## Abstract

We study estimation of the date of change in persistence, from I(0) to I(1) or vice versa. Contrary to statements in the original papers, our analytical results establish that the ratio-based break point estimators of Kim [Kim, J.Y., 2000. Detection of change in persistence of a linear time series. Journal of Econometrics 95, 97–116], Kim et al. [Kim, J.Y., Belaire-Franch, J., Badillo Amador, R., 2002. Corringendum to “Detection of change in persistence of a linear time series”. Journal of Econometrics 109, 389–392] and Busetti and Taylor [Busetti, F., Taylor, A.M.R., 2004. Tests of stationarity against a change in persistence. Journal of Econometrics 123, 33–66] are inconsistent when a mean (or other deterministic component) is estimated for the process. In such cases, the estimators converge to random variables with upper bound given by the true break date when persistence changes from I(0) to I(1). A Monte Carlo study confirms the large sample downward bias and also finds substantial biases in moderate sized samples, partly due to properties at the end points of the search interval.

## 1 Introduction

Studies of persistence change, i.e. series changing from I(0) to I(1) or vice versa, often employ ratio-based test procedures, originally proposed by Kim (2000), and further analysed by Kim et al. (2002, KBA) and Busetti and Taylor (2004, BT). This theoretical literature has focused primarily on testing the existence and nature of persistence change, apparently overlooking a problem with the associated break point estimators for the date of change. To be specific, we show that the ratio-based break point estimators of KBA (corrected from Kim, 2000) and BT are not consistent when a deterministic term (such as a mean) is estimated. The consistency established by Kim (2000) applies only in the special (and typically unrealistic) case where the process is known to have zero mean.

Therefore, consider the process^1^

(1) $$y_{t}=\beta +\mu_{t}$$

where $\mu_{t}$ is a zero mean stochastic process and β is a constant (which could be zero). A change in persistence from I(0) to I(1) can be represented by defining $\mu_{t}$ in (1) as

(2) $$\mu_{t}=\left\{ \begin{array}{cc} \varepsilon_{t} & t=1,\ldots ,\left[\tau_{0}T\right]\\ \overset{t}{\underset{s=\left[\tau_{0}T\right]+1}{\sum }}\varepsilon_{s} & t=\left[\tau_{0}T\right]+1,\ldots ,T \end{array}\right.$$

for t=1,…,T, where $\tau_{0}$ is the true break fraction, $\tau_{0}\in \left(0,1\right)$, and $\varepsilon_{t}$ is a stationary process (see Assumption 1 below).

BT propose estimating the break fraction for a persistence change from I(0) to I(1) as

(3) $${\overset{˜}{\tau }}_{BT}=\arg\underset{\tau \in \left[\tau_{l},\tau_{u}\right]}{\max}J_{BT}\left(\tau \right)$$

$$J_{BT}\left(\tau \right)=\frac{{\left[\left(1-\tau \right)T\right]}^{-2}\overset{T}{\underset{t=\left[\tau T\right]+1}{\sum }}{\overset{ˆ}{\varepsilon }}_{1,t}^{2}}{{\left[\tau T\right]}^{-2}\overset{\left[\tau T\right]}{\underset{t=1}{\sum }}{\overset{ˆ}{\varepsilon }}_{0,t}^{2}}\text{,}$$

where ${\overset{ˆ}{\varepsilon }}_{0,t}=y_{t}-{\bar{y}}_{0}$ and ${\bar{y}}_{0}$ is the sample mean computed over t=1,…,[τT], while ${\overset{ˆ}{\varepsilon }}_{1,t}=y_{t}-{\bar{y}}_{1}$ are the corresponding values for t=[τT]+1,…,T, and $\tau \in \left[\tau_{l},\tau_{u}\right]\subset \left(0,1\right)$ defines the search interval considered for the break fraction. Although they state that this estimator is also proposed independently by KBA (see BT, p.38, Remark 2.5), in fact KBA propose

(4) $${\overset{˜}{\tau }}_{KBA}=\arg\underset{\tau \in \left[\tau_{l},\tau_{u}\right]}{\max}J_{KBA}\left(\tau \right)$$

$$J_{KBA}\left(\tau \right)=\frac{{\left[\left(1-\tau \right)T\right]}^{-2}\overset{T}{\underset{t=\left[\tau T\right]+1}{\sum }}{\overset{ˆ}{\varepsilon }}_{1,t}^{2}}{{\left[\tau T\right]}^{-1}\overset{\left[\tau T\right]}{\underset{t=1}{\sum }}{\overset{ˆ}{\varepsilon }}_{0,t}^{2}}\text{.}$$

Hence

(5) $$J_{KBA}\left(\tau \right)={\left[\tau T\right]}^{-1}\;J_{BT}\left(\tau \right)$$

and since the relationship between these depends on the break fraction τ, (3) and (4) do not, in general, lead to the same estimate $\overset{˜}{\tau }$.

The next section establishes our analytical results. Specifically, we provide representations of the limiting distributions of the KBA and BT break point estimators, (3) and (4), thereby showing that these ratio-based estimators are not consistent for the true break point when mean effects are taken into account. This problem arises from the contamination of otherwise stationary sub-sample observations by subtraction of a mean that covers some nonstationary values. Analogous results apply for the estimation of the break point for a change from I(1) to I(0), as shown in the Appendix. Section 3 presents Monte Carlo results to further examine the small and large sample properties of these estimators, while Section 4 concludes.

## 2 Asymptotic results

Our key results are provided in Lemma 1 and its corollary. The conditions of Assumption 1 below permit both temporal dependence and some forms of heteroskedasticity; see Phillips (1987) and Phillips and Perron (1988).

Assumption 1 (a) $E\left[\varepsilon_{t}\right]=0$ for all t; (b) $E{\left|\varepsilon_{t}\right|}^{\gamma +\epsilon }<\infty$ for some γ>2 and ϵ>0; (c) $\varepsilon_{t}$ is α-mixing with mixing coefficients $\alpha_{m}$ that satisfy $\sum_{m=1}^{\infty }\alpha_{m}^{1-2/\gamma }<\infty$; (d) $\sigma^{2}=\lim_{T\to \infty }T^{-1}E{\left(\sum_{t=1}^{T}\varepsilon_{t}\right)}^{2}$ exists and $\sigma^{2}>0$; and (e) $\sigma_{\varepsilon }^{2}=\lim_{T\to \infty }T^{-1}\sum_{t=1}^{T}E\left[\varepsilon_{t}^{2}\right]$ is strictly positive and finite and does not depend on the break fraction $\tau_{0}$.

Lemma 1 *Suppose that the conditions of* Assumption 1 *hold and that* $\tau_{0}\in \left[\tau_{l},\tau_{u}\right]\subset \left(0,1\right)$ *in the model for a change from* I(0) *to* I(1)*, given by* (1) and (2)*. For* $J_{BT}\left(\tau \right)$ *defined in* (3) *and given* $\tau \in \left[\tau_{l},\tau_{u}\right]$

(6) $$T^{-1}J_{BT}\left(\tau \right)\Rightarrow \tau {\left(\frac{1-\tau_{0}}{1-\tau }\right)}^{2}\frac{\sigma^{2}}{\sigma_{\varepsilon }^{2}}\left[\int_{0}^{1}{\left[V\left(r\right)\right]}^{2}dr-\left(\frac{1-\tau_{0}}{1-\tau }\right){\left(\int_{0}^{1}V\left(r\right)dr\right)}^{2}\right],\tau \leq \tau_{0}$$

(7) $$J_{BT}\left(\tau \right)\Rightarrow {\left(\frac{\tau }{1-\tau }\right)}^{2}\left[\frac{\int_{\tau_{0,1}}^{1}{\left[V\left(r\right)\right]}^{2}dr-\left(\frac{1-\tau_{0}}{1-\tau }\right){\left(\int_{\tau_{0,1}}^{1}V\left(r\right)dr\right)}^{2}}{\int_{0}^{\tau_{0,1}}{\left[V\left(r\right)\right]}^{2}dr-\frac{\left(1-\tau_{0}\right)}{\tau }{\left(\int_{0}^{\tau_{0,1}}V\left(r\right)dr\right)}^{2}}\right],\tau >\tau_{0}\text{,}$$

*where* V(r) *is a standard Brownian motion on* [0,1] *and* $\tau_{0,1}=\left(\tau -\tau_{0}\right)/\left(1-\tau_{0}\right)$ *. Consequently,* ${\overset{˜}{\tau }}_{BT}$ *defined by* (3) *is not consistent since it converges to a random variable, having asymptotic upper bound of* $\tau_{0}$*.*

A proof of this lemma is provided in the Appendix. The following Corollary, relating to the KBA estimator, follows immediately, using (5).

Corollary 1 *Suppose that the conditions of* Assumption 1 *hold and that* $\tau_{0}\in \left[\tau_{l},\tau_{u}\right]\subset \left(0,1\right)$ *in the model given by* (1) and (2)*. For* $J_{KBA}\left(\tau \right)$ *defined in* (4) *and given* $\tau \in \left[\tau_{l},\tau_{u}\right]$*then*

(8) $$J_{KBA}\left(\tau \right)\Rightarrow {\left(\frac{1-\tau_{0}}{1-\tau }\right)}^{2}\frac{\sigma^{2}}{\sigma_{\varepsilon }^{2}}\left[\int_{0}^{1}{\left[V\left(r\right)\right]}^{2}dr-\left(\frac{1-\tau_{0}}{1-\tau }\right){\left(\int_{0}^{1}V\left(r\right)dr\right)}^{2}\right],\tau \leq \tau_{0}$$

(9) $$T\;J_{KBA}\left(\tau \right)\Rightarrow \frac{\tau }{{\left(1-\tau \right)}^{2}}\left[\frac{\int_{\tau_{0,1}}^{1}{\left[V\left(r\right)\right]}^{2}dr-\left(\frac{1-\tau_{0}}{1-\tau }\right){\left(\int_{\tau_{0,1}}^{1}V\left(r\right)dr\right)}^{2}}{\int_{0}^{\tau_{0,1}}{\left[V\left(r\right)\right]}^{2}dr-\frac{\left(1-\tau_{0}\right)}{\tau }{\left(\int_{0}^{\tau_{0,1}}V\left(r\right)dr\right)}^{2}}\right],\tau >\tau_{0}\text{,}$$

*where* $\tau_{0,1}$ *is defined in* Lemma 1*. Consequently,* ${\overset{˜}{\tau }}_{KBA}$ *defined by* (4) *is not consistent and converges to a random variable, having asymptotic upper bound of* $\tau_{0}$*.*

Remark 1 The representation of the asymptotic distribution for neither $J_{BT}\left(\tau \right)$ nor $J_{KBA}\left(\tau \right)$ is symmetric around $\tau_{0}$. For example, $J_{BT}\left(\tau \right)$ diverges to +∞ for $\tau \leq \tau_{0}$, while it is of $O_{p}\left(1\right)$ when $\tau >\tau_{0}$. The asymptotic representation for $J_{KBA}\left(\tau \right)$ is similarly of higher order in T for $\tau \leq \tau_{0}$ than $\tau >\tau_{0}$.

Remark 2 The inconsistency of ${\overset{˜}{\tau }}_{BT}$ and ${\overset{˜}{\tau }}_{KBA}$ for $\tau_{0}$ in (1) and (2) arises because the term in square brackets on the right-hand side of (6) and (8) is not necessarily maximised at $\tau =\tau_{0}$, due to $\left(1-\tau_{0}\right)/\left(1-\tau \right)$ being a monotonically increasing function of τ. Therefore, the maxima of these expressions varies with the specific Brownian motion process and the estimators converge to random variables.^2^ However, due to the differing orders of $J_{i}\left(\tau \right)\left(i=BT,KBA\right)$ for $\tau \leq \tau_{0}$ and $\tau >\tau_{0}$, each estimator has an asymptotic upper bound of $\tau_{0}$, implying that these ratio-based estimators are asymptotically downward biased when a mean is estimated for the process. It is anticipated that ${\overset{˜}{\tau }}_{KBA}\leq {\overset{˜}{\tau }}_{BT}$, irrespective of whether a mean is or is not estimated for the process.

Remark 3 Kim (2000, Theorem 3.5) claims to establish that ${\overset{˜}{\tau }}_{KBA}$ is a consistent estimator of $\tau_{0}$ even with deterministic terms as in (1). However, in using his Assumption 2, his proof overlooks the asymptotically non-negligible implications of mean-correction when the order of integration changes. More specifically, when $\tau <\tau_{0}$ it is invalid to assume that (in our notation) ${\overset{ˆ}{\varepsilon }}_{1,t}=y_{t}-{\bar{y}}_{1}$ is a stationary sequence over $t=\left[\tau T\right]+1,\ldots ,\left[\tau_{0}T\right]$ despite the stationarity of $y_{t}$. An analogous comment applies for the case $\tau >\tau_{0}$; in particular, stationarity does not hold for ${\overset{ˆ}{\varepsilon }}_{0,t}=y_{t}-{\overset{¯}{y}}_{0},t=\left[\tau_{0}T\right]+1,\ldots ,\left[\tau T\right]$ because I(1) observations enter ${\overset{¯}{y}}_{0}$.^3^

Remark 4 When (1) contains no deterministic component and no mean effect is estimated, the second term in square brackets in (6)–(9) does not appear. For this special case, (6) and (8) are both maximised at $\tau =\tau_{0}$ and both estimators are consistent for the true change point.

Remark 5 Each expression (6)–(9) has a denominator factor (1−τ), which may give rise to bimodality as $\tau \to \tau_{u}$, when $\tau_{u}$ is relatively close to 1.

Remark 6 For $\tau >\tau_{0}$, but τ approaching $\tau_{0}$ from above, then both $\int_{0}^{\tau_{0,1}}{\left[V\left(r\right)\right]}^{2}dr$ and $\int_{0}^{\tau_{0,1}}V\left(r\right)dr\to 0$ where $\tau_{0,1}=\left(\tau -\tau_{0}\right)/\left(1-\tau_{0}\right)$. Thus, $J_{i}\left(\tau \right)\;\left(i=BT,KBA\right)$ diverge to +∞, so that (7), and (9) might be maximised on $\tau >\tau_{0}$ when $\tau \to \tau_{0}$. For the integral and consequently for the statistics to be well defined, $\tau -\tau_{0}$ should not be close to zero. In practice, however, it is usual to consider all available observations as potential break points and hence the computed value of the denominator may be very small, leading to maximisation of the statistics for observations immediately subsequent to (rather than at) the true break point.

When considering a change in persistence from I(1) to I(0), the roles of the two subsamples are interchanged from those considered in Lemma 1 and Corollary 1. These results are provided in the Appendix.

## 3 Monte Carlo evidence

This section uses Monte Carlo simulations to investigate the properties of the BT and KBA estimators for a change-point in persistence from I(0) to I(1) for a range of sample sizes.^4^ The data generation process (DGP) is given in (1) and (2) with an intercept included in the regression, specifically $\beta =5,\varepsilon_{t}∼N\left(0,1\right)$ and the true break fractions are given by $\tau_{0}=\left\{0.3,\;0.5,\;0.7\right\}$. For all cases we generate series of T={100,1000,5000,10,000,20,000} observations and a total of 10,000 replications are carried out for each design. The discussion below is divided into two subsections, the first examining “small” sample properties and the second “large” sample ones, with the latter providing evidence on the asymptotic properties of the ratio-based estimators considered in the preceding section.

Although the DGP always exhibits a change in the order of integration, we follow empirical practice and employ a pre-test for the presence of a change in persistence using the sup-type test of BT (2004) at the 5% significance level. Only replications for which a break in persistence is detected are retained for estimating the break fractions.^5^ The tests and estimation of the change points apply the search interval τ∈[0.2,0.8].

### 3.1 Small sample properties

**Fig. 1:**
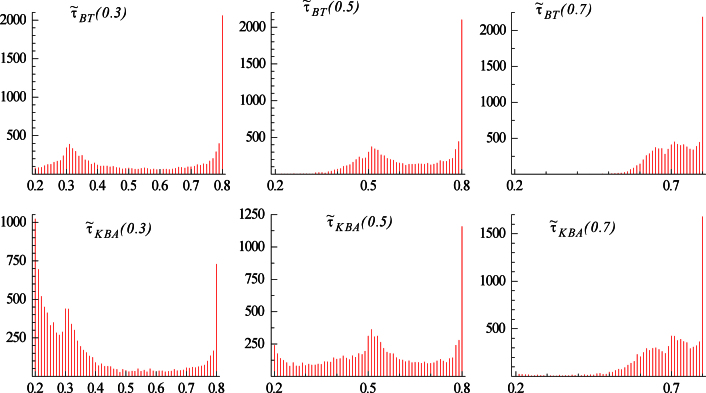
Distribution of break fraction estimators for a change from I(0) to I(1) for T=100.

**Fig. 2:**
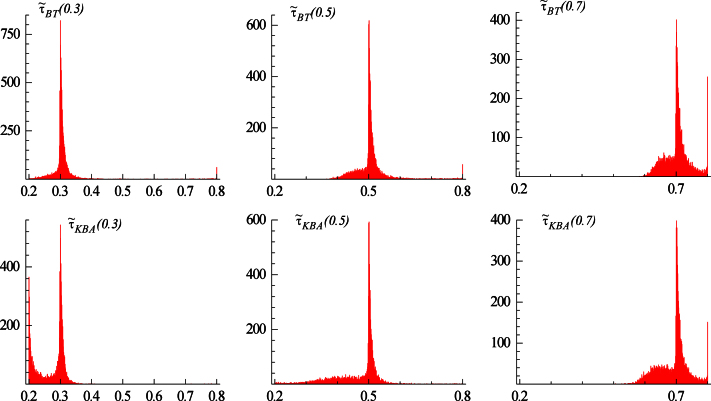
Distribution of break fraction estimators for change from I(0) to I(1) for T=1000.

The sample mean and mean absolute deviation of the break fractions for a change from I(0) to I(1) are reported in Table 1 where (as usual in this literature) the search considers every observation within the range $\left[\tau_{l}T,\tau_{u}T\right]=\left[0.2T,\;0.8T\right]$ as the potential break point. The resulting empirical distributions, for sample sizes of 100 and 1000 observations, are also presented in Figs. 1 and 2.

The finite sample results for the BT and KBA estimators shown in Table 1 appear to be the consequence of two partially off-setting effects noted in Section 2, namely the upward bias from the bimodality of the distributions commented on in Remark 5 and the mean-correction resulting in an asymptotic downward bias as noted in Remark 2. For the BT estimator, the former effect is the stronger, which is evident in the upper-boundary estimates seen in Fig. 1 with T=100. This clustering of ${\overset{˜}{\tau }}_{BT}$ at $\tau_{u}=0.8$ occurs even when $\tau_{0}=0.3$. This also explains why the bias for this estimator in Table 1 is less severe for larger values of $\tau_{0}$. Although the properties improve as the sample size increases, the BT estimator nevertheless leads to clustering at the upper limit even with T=1000 which is especially noticeable in Fig. 2 when $\tau_{0}=0.7$. On the other hand, for the KBA estimator, and particularly as the sample size increases, the asymptotic downward bias becomes the stronger effect, although the KBA estimator also suffers from bimodality at the upper limit, which similarly does not disappear even for T=1000. Also note that the KBA estimator exhibits a peak at the lower limit of $\tau_{l}=0.2$ when $\tau_{0}=0.3$, which remains perceptible in Fig. 2 for T=1000. Further, the means of the estimates always exhibit the ordering ${\overset{˜}{\tau }}_{KBA}\leq {\overset{˜}{\tau }}_{BT}$ anticipated in Remark 2.

### 3.2 Large sample properties

Table 1 sheds only limited evidence about the asymptotic properties of the ratio-based estimators when mean corrections are applied. For example, as T increases, the means of the BT and KBA are decreasing, but the mean of ${\overset{˜}{\tau }}_{BT}$ is greater than $\tau_{0}$ with T=1000, and hence does not exhibit the anticipated asymptotic upper bound of $\tau_{0}$ in Lemma 1. To investigate the empirical large sample behaviour of these estimators for the non-zero mean case, Table 2 presents results for samples of T=5000,10,000,20,000.^6^ The results in this table also show, for each case, the percentage of replications for which the estimate coincides with the true value and the percentage of replications for which the estimate exceeds $\tau_{0}$ (denoted %True and %After, respectively). The latter is included to investigate the implication of the theoretical analysis that $\tau_{0}$ provides an asymptotic upper bound for the estimated break fraction using the BT or KBA estimators.

Table 2 supports the analytical results. Each ratio-based estimator is downward biased, with a mean that is effectively independent of T for these large sample sizes. Further, since the relationship ${\overset{˜}{\tau }}_{KBA}\leq {\overset{˜}{\tau }}_{BT}$ also applies (see Remark 2), the KBA estimator suffers greater large sample biases than the BT estimator. In particular, when mean effects are allowed, the KBA estimator is downward biased by around 3% when $\tau_{0}=0.3$, with average ${\overset{˜}{\tau }}_{KBA}$ of around 0.27, with the bias being a little larger when $\tau_{0}=0.5$. The biases for both estimators in Table 2 are very similar to the biases shown for a sample size of T=1000 in Table 1. However, irrespective of the particular estimator and the true break fraction $\tau_{0}$, the mean absolute deviations barely change with T in Table 2, providing evidence of the asymptotic random nature of the estimators. Finally, with these large sample sizes, only a small (and declining) percentage of estimates exceed the true $\tau_{0}$ supporting the theoretical result that this true value provides an asymptotic upper bound for the random BT and KBA break fraction estimators (see Lemma 1 and Corollary 1). Indeed, these results further emphasise the poor performance of the KBA estimator when $\tau_{0}=0.3$, with 20% of the estimates here being at the lower boundary of the search interval, irrespective of T=5000,10,000 or 20,000.

## 4 Conclusion

This paper shows analytically that the ratio-based break fraction estimators of BT and KBA are not consistent for the true break point when mean effects have to be taken into account through a prior regression. To be specific, both estimators converge to random variables which have upper bound equal to the true break fraction and hence exhibit large sample downward biases when persistence changes from I(0) to I(1). A Monte Carlo analysis shows that the KBA change point estimator can show substantial biases for all sample sizes when mean-corrected residuals are employed. In relatively small samples, this results from a combination of clustering of estimates at the upper bound of the search interval together with the off-setting effects due to the lack of consistency of the estimator which has an asymptotic upper bound of $\tau_{0}$. Our simulations imply that, for at least some values of $\tau_{0}$, the latter effect outweighs the former. Although it also suffers from a lack of consistency, the BT estimator has less severe large sample bias relative to the KBA estimator, but it appears to be badly upward biased in small samples due to the bimodality at the upper bound of the search interval, which is not compensated by the (small sample) effects of a substantial asymptotic downward bias.

Finally, it should be noted that the lack of consistency of the KBA and BT break fraction estimators is not shared by the estimator of Leybourne et al. (2006). That break point estimator for a change in persistence is based on a scaled cumulated sum of squares and does not give rise to the problem studied here.

## Figures and Tables

**Table 1:** Empirical properties of break fraction estimators for I(0) to I(1) for small/moderate sample sizes.

	$\tau_{0}=0.3$		$\tau_{0}=0.5$		$\tau_{0}=0.7$	
	${\overset{˜}{\tau }}_{BT}$	${\overset{˜}{\tau }}_{KBA}$	${\overset{˜}{\tau }}_{BT}$	${\overset{˜}{\tau }}_{KBA}$	${\overset{˜}{\tau }}_{BT}$	${\overset{˜}{\tau }}_{KBA}$
T=100						
Mean	0.450	0.329	0.579	0.486	0.721	0.686
Abs. Dev.	0.171	0.104	0.119	0.141	0.062	0.080
T=1000						
Mean	0.311	0.272	0.505	0.468	0.704	0.691
Abs. Dev.	0.020	0.036	0.027	0.047	0.028	0.034

Notes: The results are based on 10,000 replications, with the break fraction estimated for replications in which the null hypothesis of constant order of integration is rejected at the 5% level. Tests and break fraction estimation employ searches over all observations in the interval τ∈[0.2,0.8].

**Table 2:** Empirical large sample properties of ratio-based break fraction estimators for I(0) to I(1).

	$\tau_{0}=0.3$		$\tau_{0}=0.5$		$\tau_{0}=0.7$	
	${\overset{˜}{\tau }}_{BT}$	${\overset{˜}{\tau }}_{KBA}$	${\overset{˜}{\tau }}_{BT}$	${\overset{˜}{\tau }}_{KBA}$	${\overset{˜}{\tau }}_{BT}$	${\overset{˜}{\tau }}_{KBA}$
T=5000						
Mean	0.296	0.269	0.489	0.465	0.689	0.680
Abs. Dev.	0.004	0.031	0.011	0.036	0.012	0.020
%Extreme	0.02	19.2	0.01	0	0.03	0
%True	84.5	58.3	71.4	60.0	68.9	63.3
%After	2.60	0.37	4.51	1.84	3.62	2.10
T=10,000						
Mean	0.296	0.269	0.490	0.465	0.689	0.680
Abs. Dev.	0.004	0.031	0.010	0.035	0.012	0.020
%Extreme	0	19.7	0	0	0	0
%True	87.7	60.4	75.8	62.3	67.3	61.5
%After	0.14	0	0.65	0.11	2.85	1.67
T=20,000						
Mean	0.296	0.270	0.490	0.468	0.688	0.680
Abs. Dev.	0.004	0.030	0.009	0.032	0.011	0.020
%Extreme	0	20.8	0	0	0	0
% True	88.0	62.0	77.9	63.5	70.8	64.0
%After	0	0	0.01	0	0.27	0.11

Notes: as for Table 1, except that break fraction estimation is conducted by searching in steps of 0.01 within the interval τ∈[0.2,0.8], with %Extreme being the percentage of break fraction estimates that lie at an end-point of the search interval, and %True and %After being the percentages of estimates that are equal to and exceed $\tau_{0}$, respectively.

## Appendix

The first part of the appendix provides a proof of Lemma 1, while the second part discusses a change in persistence from I(1) toI(0).

Proof of Lemma 1 For a change from I(0) to I(1), rewrite the BT statistic of (3) as

(10) $$J_{BT}\left(\tau \right)=\frac{\left[\tau T\right]{\left[\left(1-\tau \right)T\right]}^{-2}\overset{T}{\underset{t=\left[\tau T\right]+1}{\sum }}{\overset{ˆ}{\varepsilon }}_{1,t}^{2}}{{\left[\tau T\right]}^{-1}\overset{\left[\tau T\right]}{\underset{t=1}{\sum }}{\overset{ˆ}{\varepsilon }}_{0,t}^{2}}\text{.}$$

Consider first $\tau =\tau_{0}$. For the denominator, ${\left(\left[\tau_{0}T\right]\right)}^{-1}\sum_{t=1}^{\left[\tau_{0}T\right]}{\overset{ˆ}{\varepsilon }}_{0,t}^{2}\overset{p}{⟶}\sigma_{\varepsilon }^{2}$. For the numerator, define $T_{1}=\left[\left(1-\tau_{0}\right)T\right]$ and a functional central limit theorem (FCLT) yields

(11) $$T_{1}^{\;-1/2}\mu_{\left[rT\right]}\Rightarrow \sigma \left[V\left(r_{1}\right)\right]\text{,}\;r_{1}=\left(r-\tau_{0}\right)/\left(1-\tau_{0}\right),\;r\in \left(\tau_{0},1\right]$$

where V(⋅) is a standard Brownian motion on [0,1], since (2) defines a zero-mean I(1) process for $\mu_{\left[rT\right]},r>\tau_{0}$, with starting value $\mu_{\left[\tau_{0}T\right]}=0$. From (11),

$$T_{1}^{\;-1/2}\;{\bar{\mu }}_{1}=T_{1}^{\;-3/2}\overset{T}{\underset{t=\left[\tau_{0}T\right]+1}{\sum }}\mu_{t}\Rightarrow \sigma {\left(1-\tau_{0}\right)}^{-1}\int_{\tau_{0}}^{1}V\left(\frac{s-\tau_{0}}{1-\tau_{0}}\right)ds=\sigma \;\left[\int_{0}^{1}V\left(s_{1}\right)ds_{1}\right]$$

where the result in the second line follows by the continuous mapping theorem (CMT) and the last line follows by a change of variable $s_{1}=\frac{s-\tau_{0}}{1-\tau_{0}}\in \left(0,1\right)$. Hence

(12) $$T_{1}^{\;-1/2}{\overset{ˆ}{\varepsilon }}_{1,\left[rT\right]}=T_{1}^{\;-1/2}\left[\mu_{\left[rT\right]}-{\bar{\mu }}_{1}\right]\;r\in \left(\tau_{0},1\right]\Rightarrow \sigma \left(V\left(r_{1}\right)-\int_{0}^{1}V\left(s_{1}\right)ds_{1}\right)\;r_{1}=\left(r-\tau_{0}\right)/\left(1-\tau_{0}\right)\text{.}$$

Thus

(13) $$T_{1}^{\;-2}\overset{T}{\underset{t=\left[\tau_{0}T\right]+1}{\sum }}{\overset{ˆ}{\varepsilon }}_{1,t}^{2}\Rightarrow \sigma^{2}\int_{0}^{1}{\left(V\left(r_{1}\right)-\int_{0}^{1}V\left(s_{1}\right)ds_{1}\right)}^{2}dr_{1}$$

so that

$$\frac{1}{T}J_{BT}\left(\tau_{0}\right)\Rightarrow \tau_{0}\frac{\sigma^{2}}{\sigma_{\varepsilon }^{2}}\left[\int_{0}^{1}{\left(V\left(r_{1}\right)\right)}^{2}dr_{1}-{\left\{\int_{0}^{1}V\left(s_{1}\right)ds_{1}\right\}}^{2}\right]$$

which yields that $J_{BT}\left(\tau_{0}\right)$ diverges to +∞ as T→∞. Now consider the case of a given $\tau <\tau_{0}$. Retaining the definition $T_{1}=\left[\left(1-\tau_{0}\right)T\right]$, then

(14) $$T_{1}^{\;-1/2}\;{\bar{\mu }}_{1}=T_{1}^{\;-1/2}{\left[\left(1-\tau \right)T\right]}^{-1}\left\{\overset{T}{\underset{t=\left[\tau_{0}T\right]+1}{\sum }}\mu_{t}+\overset{\left[\tau_{0}T\right]}{\underset{t=\left[\tau T\right]+1}{\sum }}\varepsilon_{t}\right\}\Rightarrow \sigma \left(\frac{1-\tau_{0}}{1-\tau }\right)\;\int_{0}^{1}V\left(s_{1}\right)ds_{1}$$

and hence

$$T_{1}^{\;-1/2}{\overset{ˆ}{\varepsilon }}_{1,\left[rT\right]}\Rightarrow \left\{ \begin{array}{c} \sigma \left(V\left(r_{1}\right)-\left(\frac{1-\tau_{0}}{1-\tau }\right)\;\int_{0}^{1}V\left(s_{1}\right)ds_{1}\right)\text{,}\;r_{1}=\left(r-\tau_{0}\right)/\left(1-\tau_{0}\right),\;r\in \left(\tau_{0},1\right]\\ -\sigma \left(\frac{1-\tau_{0}}{1-\tau }\right)\;\int_{0}^{1}V\left(s_{1}\right)ds_{1}\text{,}\;r\in \left(\tau ,\tau_{0}\right]\text{.} \end{array}\right.$$

Therefore,

(15) $$T_{1}^{\;-2}\overset{T}{\underset{t=\left[\tau T\right]+1}{\sum }}{\overset{ˆ}{\varepsilon }}_{1,t}^{2}=T_{1}^{\;-2}\left[\overset{\left[\tau_{0}T\right]}{\underset{t=\left[\tau T\right]+1}{\sum }}{\overset{ˆ}{\varepsilon }}_{1,t}^{2}+\overset{T}{\underset{t=\left[\tau_{0}T\right]+1}{\sum }}{\overset{ˆ}{\varepsilon }}_{1,t}^{2}\right]\Rightarrow \sigma^{2}\left[\int_{0}^{1}{\left[V\left(r_{1}\right)\right]}^{2}dr_{1}-\left(\frac{1-\tau_{0}}{1-\tau }\right){\left\{\int_{0}^{1}V\left(r_{1}\right)dr_{1}\right\}}^{2}\right]\text{.}$$

Moreover, for the denominator of $J_{BT}\left(\tau \right)$

(16) $${\left[\tau T\right]}^{-1}\overset{\left[\tau T\right]}{\underset{t=1}{\sum }}{\overset{ˆ}{\varepsilon }}_{0,t}^{2}\overset{p}{⟶}\sigma_{\varepsilon }^{2}\text{.}$$

Substituting from (15) and (16) into (10) yields the required expression (6) for $\tau \leq \tau_{0}$. Finally, consider a given $\tau >\tau_{0}$. Firstly, since $y_{t}∼I\left(1\right)$ for $t>\left[\tau_{0}T\right]$, then (11) continues to hold for r∈(τ,1], while

$$T_{1}^{\;-1/2}\;{\bar{\mu }}_{1}\Rightarrow \sigma \;\left(\frac{1-\tau_{0}}{1-\tau }\right)\left[\int_{\tau_{0,1}}^{1}\left[V\left(s_{1}\right)\right]ds_{1}\right]\text{,}\;\tau_{0,1}=\left(\tau -\tau_{0}\right)/\left(1-\tau_{0}\right)\text{.}$$

Therefore, (12) is replaced by

(17) $$T_{1}^{\;-1/2}{\overset{ˆ}{\varepsilon }}_{1,\left[rT\right]}\Rightarrow \sigma \left[V\left(r_{1}\right)-\left(\frac{1-\tau_{0}}{1-\tau }\right)\left(\int_{\tau_{0,1}}^{1}V\left(s_{1}\right)ds_{1}\right)\right]\text{,}\;r_{1}=\left(r-\tau_{0}\right)/\left(1-\tau_{0}\right)$$

for r∈(τ,1] and, in relation to the numerator, using the CMT

(18) $$T_{1}^{\;-2}\overset{T}{\underset{t=\left[\tau T\right]+1}{\sum }}{\overset{ˆ}{\varepsilon }}_{1,t}^{2}\Rightarrow \sigma^{2}\left[\int_{\tau_{0,1}}^{1}{\left[V\left(r_{1}\right)\right]}^{2}dr_{1}-\left(\frac{1-\tau_{0}}{1-\tau }\right){\left\{\int_{\tau_{0,1}}^{1}V\left(s_{1}\right)ds_{1}\right\}}^{2}\right]$$

since $\int_{\tau_{0,1}}^{1}1\;dr_{1}=1-\tau_{0,1}=\left(1-\tau \right)/\left(1-\tau_{0}\right)$. Secondly, the denominator of the statistic is

$${\left[\tau T\right]}^{-2}\overset{\left[\tau T\right]}{\underset{t=1}{\sum }}{\overset{ˆ}{\varepsilon }}_{0,t}^{2}={\left[\tau T\right]}^{-2}\left(\overset{\left[\tau_{0}T\right]}{\underset{t=1}{\sum }}{\overset{ˆ}{\varepsilon }}_{0,t}^{2}+\overset{\left[\tau T\right]}{\underset{t=\left[\tau_{0}T\right]+1}{\sum }}{\overset{ˆ}{\varepsilon }}_{0,t}^{2}\right)$$

in which

$$T_{1}^{\;-1/2}\;{\bar{\mu }}_{0}=T_{1}^{\;-1/2}{\left[\tau T\right]}^{-1}\left\{\overset{\left[\tau_{0}T\right]}{\underset{t=1}{\sum }}\varepsilon_{t}+\overset{\left[\tau T\right]}{\underset{t=\left[\tau_{0}T\right]+1}{\sum }}\mu_{t}\right\}\Rightarrow \sigma \;\left(\frac{1-\tau_{0}}{\tau }\right)\int_{0}^{\tau_{0,1}}V\left(s_{1}\right)ds_{1}$$

where $\tau_{0,1}$ is defined as above. Further, $T_{1}^{\;-1/2}\mu_{\left[rT\right]}\Rightarrow \sigma \left[V\left(r_{1}\right)\right]$ for $r_{1}=\left(r-\tau_{0}\right)/\left(1-\tau_{0}\right)$ and $r>\tau_{0}$. Thus

$$T_{1}^{\;-1/2}{\overset{ˆ}{\varepsilon }}_{0,\left[rT\right]}\Rightarrow \left\{ \begin{array}{c} \sigma \left[V\left(r_{1}\right)-\left(\frac{1-\tau_{0}}{\tau }\right)\int_{0}^{\tau_{0,1}}V\left(s_{1}\right)ds_{1}\right]\text{,}\;r\in \left(\tau_{0},\tau \right]\\ -\sigma \;\left(\frac{1-\tau_{0}}{\tau }\right)\int_{0}^{\tau_{0,1}}V\left(s_{1}\right)ds_{1}\text{,}\;r\in \left[0,\tau_{0}\right]\text{.} \end{array}\right.$$

By the CMT

(19) $$T_{1}^{\;-2}\overset{\left[\tau T\right]}{\underset{t=1}{\sum }}{\overset{ˆ}{\varepsilon }}_{0,t}^{2}\Rightarrow \sigma^{2}\left[\int_{0}^{\tau_{0,1}}{\left[V\left(r_{1}\right)\right]}^{2}dr_{1}-\frac{1-\tau_{0}}{\tau }{\left\{\int_{0}^{\tau_{0,1}}V\left(s_{1}\right)ds_{1}\right\}}^{2}\right]\text{.}$$

From (19) and (13), the representation of the limit distribution of $J_{BT}\left(\tau \right)$ for $\tau >\tau_{0}$ is then given as in (7). Further, for $\tau >\tau_{0}$, (7) implies that

$$\underset{\tau \in \left[\tau_{l},\tau_{u}\right]}{\sup}{\left(\frac{\tau }{1-\tau }\right)}^{2}\left[\frac{\int_{\tau_{0,1}}^{1}{\left[V\left(r\right)\right]}^{2}dr-\left(\frac{1-\tau_{0}}{1-\tau }\right){\left(\int_{\tau_{0,1}}^{1}V\left(r\right)dr\right)}^{2}}{\int_{0}^{\tau_{0,1}}{\left[V\left(r\right)\right]}^{2}dr-\frac{\left(1-\tau_{0}\right)}{\tau }{\left(\int_{0}^{\tau_{0,1}}V\left(r\right)dr\right)}^{2}}\right]=O_{p}\left(1\right)\text{.}$$

Combining results for $\tau \leq \tau_{0}$ and $\tau >\tau_{0}$, the joint convergence result follows from arguments similar to those in Zivot and Andrews (1992). Writing

$$\underset{\tau \in \left[\tau_{l},\tau_{u}\right]}{\sup}T^{-1}J_{BT}\left(\tau \right)=\underset{\tau \in \left[\tau_{l},\tau_{u}\right]}{\sup}\left[T^{-1}J_{BT}\left(\tau \right)1\left(\tau \leq \tau_{0}\right)+T^{-1}J_{BT}\left(\tau \right)1\left(\tau >\tau_{0}\right)\right]$$

where 1(⋅) is an indicator function taking the value unity when the expression in parentheses is satisfied, then

(20) $$\underset{\tau \in \left[\tau_{l},\tau_{u}\right]}{\sup}T^{-1}J_{BT}\left(\tau \right)-\underset{\tau \in \left[\tau_{l},\tau_{0}\right]}{\sup}T^{-1}J_{BT}\left(\tau \right)\leq \underset{\tau \in \left(\tau_{0},\tau_{u}\right]}{\sup}T^{-1}J_{BT}\left(\tau \right)$$

and firstly, we establish that $\sup_{\tau \in \left(\tau_{0},\tau_{u}\right]}T^{-1}J_{BT}\left(\tau \right)=o_{p}\left(1\right)$. Specifically, denoting as $o_{p,\tau }\left(1\right)$ any random variable ς(τ) such that $\sup_{\tau \in \left[\tau_{l},\tau_{u}\right]}\left|\varsigma \left(\tau \right)\right|\overset{p}{⟶}0$, then

$$\underset{\tau \in \left(\tau_{0},\tau_{u}\right]}{\sup}J_{BT}\left(\tau \right)=\underset{\tau \in \left(\tau_{0},\tau_{u}\right]}{\sup}\frac{{\left[\left(1-\tau \right)T\right]}^{-2}\overset{T}{\underset{t=\left[\tau T\right]+1}{\sum }}\overset{2}{\underset{1,t}{\overset{ˆ}{\varepsilon }}}}{{\left[\tau T\right]}^{-2}\left(\overset{\left[\tau_{0}T\right]}{\underset{t=1}{\sum }}\overset{2}{\underset{0,t}{\overset{ˆ}{\varepsilon }}}+\overset{\left[\tau T\right]}{\underset{t=\left[\tau_{0}T\right]+1}{\sum }}\overset{2}{\underset{0,t}{\overset{ˆ}{\varepsilon }}}\right)}=\underset{\tau \in \left(\tau_{0},\tau_{u}\right]}{\sup}\frac{\tau^{2}}{{\left(1-\tau \right)}^{2}}T^{-1}\overset{T}{\underset{t=\left[\tau T\right]+1}{\sum }}{\left(\sigma^{-1}\overset{-1/2}{\underset{1}{T}}\mu_{t}-{\left[\left(1-\tau \right)T\right]}^{-1}\overset{T}{\underset{t=\left[\tau T\right]+1}{\sum }}\sigma^{-1}\overset{-1/2}{\underset{1}{T}}\mu_{t}\right)}^{2}{\left[T^{-1}\overset{\left[\tau_{0}T\right]}{\underset{t=1}{\sum }}{\left(-{\left[\tau T\right]}^{-1}\overset{\left[\tau T\right]}{\underset{t=\left[\tau_{0}T\right]+1}{\sum }}\sigma^{-1}\overset{-1/2}{\underset{1}{T}}\mu_{t}\right)}^{2}+T^{-1}\overset{\left[\tau T\right]}{\underset{t=\left[\tau_{0}T\right]+1}{\sum }}{\left(\sigma^{-1}\overset{-1/2}{\underset{1}{T}}\mu_{t}-{\left[\tau T\right]}^{-1}\overset{\left[\tau T\right]}{\underset{t=\left[\tau_{0}T\right]+1}{\sum }}\sigma^{-1}\overset{-1/2}{\underset{1}{T}}\mu_{t}\right)}^{2}\right]}^{-1}+o_{p,\tau }\left(1\right)=g_{1}\left(X_{T},\left[\tau T\right]/T\right)+o_{p,\tau }\left(1\right)=h^{*}\left(h_{1}\left[c_{1}\left[\left[\tau T\right]/T\right],H_{1}\left[X_{T,}\left[\tau T\right]/T\right],H_{2}\left[X_{T},\left[\tau T\right]/T\right],H_{3}\left[X_{T},\left[\tau T\right]/T\right]\right]\right)+o_{p,\tau }\left(1\right)\text{,}$$

where $X_{T}\left(r\right)=\sigma^{-1}T_{1}^{-1/2}\mu_{\left[rT\right]},r\in \left(\tau_{0},1\right]$, is the standardised empirical process. In particular, $h^{*}\left(m\right)=\sup_{\tau \in \left(\tau_{0},\tau_{u}\right]}m\left(\tau \right)$ maps a function on $\tau \in \left(\tau_{0},\tau_{u}\right]$ into the positive real line, $h_{1}$ maps four functions on $\left(\tau_{0},\tau_{u}\right]$ into a function on $\left(\tau_{0},\tau_{u}\right]$

$$h_{1}\left[m_{1},m_{2},m_{3,}m_{4}\right]\left(\cdot \right)=m_{1}\left(\cdot \right)m_{2}\left(\cdot \right){\left[m_{3}\left(\cdot \right)+m_{4}\left(\cdot \right)\right]}^{-1}\text{.}$$

Further, $c_{1}\left(\cdot \right)={\left(\tau /\left(1-\tau \right)\right)}^{2}$ maps a function on $\left(\tau_{0},\tau_{u}\right]$ into a function on $\left(\tau_{0},\tau_{u}\right]$, and $H_{1},H_{2}$ and $H_{3}$ map functions on $\left[0,1\right]\times \left(\tau_{0},\tau_{u}\right]$ into functions on $\left(\tau_{0},\tau_{u}\right]$ and are functional analogues of the standardised empirical process, specifically

$$T^{-1}\overset{T}{\underset{t=\left[\tau T\right]+1}{\sum }}{\left(\sigma^{-1}T_{1}^{-1/2}\mu_{t}-{\left[\left(1-\tau \right)T\right]}^{-1}\overset{T}{\underset{t=\left[\tau T\right]+1}{\sum }}\sigma^{-1}T_{1}^{-1/2}\mu_{t}\right)}^{2}=\int_{\tau }^{1}{\left(X_{T}\left(r_{1}\right)-{\left(1-\tau \right)}^{-1}\int_{\tau }^{1}X_{T}\left(s_{1}\right)ds\right)}^{2}dr+o_{p,\tau }\left(1\right)=H_{1}\left[X_{T},\left[\tau T\right]/T\right]+o_{p,\tau }\left(1\right)\text{,}$$

$$T^{-1}\overset{\left[\tau_{0}T\right]}{\underset{t=1}{\sum }}{\left(-{\left[\tau T\right]}^{-1}\overset{\left[\tau T\right]}{\underset{t=\left[\tau_{0}T\right]+1}{\sum }}\sigma^{-1}T_{1}^{-1/2}\mu_{t}\right)}^{2}=-\frac{1-\tau_{0}}{\tau^{2}}{\left(\int_{\tau_{0}}^{\tau }X_{T}\left(s_{1}\right)ds\right)}^{2}+o_{p,\tau }\left(1\right)=H_{2}\left[X_{T},\left[\tau T\right]/T\right]+o_{p,\tau }\left(1\right)$$

 and

$$T^{-1}\overset{\left[\tau T\right]}{\underset{t=\left[\tau_{0}T\right]+1}{\sum }}{\left(\sigma^{-1}T_{1}^{-1/2}\mu_{t}-{\left[\tau T\right]}^{-1}\overset{\left[\tau T\right]}{\underset{t=\left[\tau_{0}T\right]+1}{\sum }}\sigma^{-1}T_{1}^{-1/2}\mu_{t}\right)}^{2}=\int_{\tau_{0}}^{\tau }\left(V\left(r_{1}\right)-\tau^{-1}\int_{\tau_{0}}^{\tau }X_{T}\left(s_{1}\right)ds\right)dr+o_{p,\tau }\left(1\right)=H_{3}\left[X_{T},\left[\tau T\right]/T\right]+o_{p,\tau }\left(1\right)\text{.}$$

Thus, the continuity of $g_{1}\left(\cdot \right)$ follows from a composition of continuous functions. Given the joint convergence of $\left(X_{T}\left(\cdot \right),\left[\tau T\right]/T\right)\Rightarrow \left(V\left(r_{1}\right),\tau \right),r_{1}=\frac{r-\tau_{0}}{1-\tau_{0}},r\in \left(\tau_{0},1\right]$, and applying the CMT, the representation of the limit distribution of $J_{BT}\left(\tau \right)$ for $\tau >\tau_{0}$ as given in (7) holds uniformly over τ. This establishes that $\sup_{\tau \in \left(\tau_{0},\tau_{u}\right]}T^{-1}J_{BT}\left(\tau \right)=T^{-1}O_{p}\left(1\right)=o_{p}\left(1\right)$. Therefore, from (20)

$$\underset{\tau \in \left[\tau_{l},\tau_{u}\right]}{\sup}T^{-1}J_{BT}\left(\tau \right)-\underset{\tau \in \left[\tau_{l},\tau_{0}\right]}{\sup}T^{-1}J_{BT}\left(\tau \right)=o_{p}\left(1\right)\text{.}$$

Let ${\overset{ˆ}{\sigma }}_{\varepsilon }^{2}={\left[\tau T\right]}^{-1}\sum_{t=1}^{\left[\tau T\right]}{\overset{ˆ}{\varepsilon }}_{0,t}^{2}$ and denoting as $o_{p,\tau }\left(1\right)$ any random variable ς(τ) such that $\sup_{\tau \in \left[\tau_{l},\tau_{u}\right]}\left|\varsigma \left(\tau \right)\right|\overset{p}{⟶}0$, then the BT test statistic can be written as in Box I Box I

$$\underset{\tau \in \left[\tau_{l},\tau_{u}\right]}{\sup}T^{-1}J_{BT}\left(\tau \right)=\underset{\tau \in \left[\tau_{l},\tau_{0}\right]}{\sup}\frac{\tau \left(1-\tau_{0}\right)T^{-1}\left(\overset{\left[\tau_{0}T\right]}{\underset{t=\left[\tau T\right]+1}{\sum }}{\left(\overset{-1/2}{\underset{1}{T}}{\overset{ˆ}{\varepsilon }}_{1,t}\right)}^{2}+\overset{T}{\underset{t=\left[\tau_{0}T\right]+1}{\sum }}{\left(\overset{-1/2}{\underset{1}{T}}{\overset{ˆ}{\varepsilon }}_{1,t}\right)}^{2}\right)}{{\left(1-\tau \right)}^{2}\overset{2}{\underset{\varepsilon }{\overset{ˆ}{\sigma }}}}+o_{p,\tau }\left(1\right)=\underset{\tau \in \left[\tau_{l},\tau_{0}\right]}{\sup}\frac{\tau \left(1-\tau_{0}\right)}{{\left(1-\tau \right)}^{2}}\frac{\sigma^{2}}{\overset{2}{\underset{\varepsilon }{\overset{ˆ}{\sigma }}}}\left[T^{-1}\overset{\left[\tau_{0}T\right]}{\underset{t=\left[\tau T\right]+1}{\sum }}{\left(-{\left[\left(1-\tau \right)T\right]}^{-1}\overset{T}{\underset{t=\left[\tau T\right]+1}{\sum }}\overset{-1/2}{\underset{1}{T}}\sigma^{-1}\mu_{t}\right)}^{2}+T^{-1}\overset{T}{\underset{t=\left[\tau_{0}T\right]+1}{\sum }}{\left(\overset{-1/2}{\underset{1}{T}}\sigma^{-1}\mu_{t}-{\left[\left(1-\tau \right)T\right]}^{-1}\overset{T}{\underset{t=\left[\tau T\right]+1}{\sum }}\overset{-1/2}{\underset{1}{T}}\sigma^{-1}\mu_{t}\right)}^{2}\right]+o_{p,\tau }\left(1\right)=g_{2}\left(X_{T},\overset{2}{\underset{\varepsilon }{\overset{ˆ}{\sigma }}},\sigma^{2},\left[\tau T\right]/T\right)+o_{p,\tau }\left(1\right)=h^{*}\left(h_{2}\left[\sigma^{2},\overset{2}{\underset{\varepsilon }{\overset{ˆ}{\sigma }}},c_{2}\left[\left[\tau T\right]/T\right],H_{4}\left[X_{T},\left[\tau T\right]/T\right],H_{5}\left[X_{T},\left[\tau T\right]/T\right]\right]\right)+o_{p,\tau }\left(1\right)\text{.}$$

. Similarly as above, continuity of $g_{2}\left(\cdot \right)$ is established from continuity of composition functions, where $h_{2}$ maps two positive real numbers and three functions on $\left[\tau_{l},\tau_{0}\right]$ into a function on $\left[\tau_{l},\tau_{0}\right]$

$$h_{2}\left[m_{1},m_{2},m_{3},m_{4},m_{5}\right]\left(\cdot \right)=m_{1}\left(\cdot \right)m_{2}{\left(\cdot \right)}^{-1}m_{3}\left(\cdot \right)\left[m_{4}\left(\cdot \right)+m_{5}\left(\cdot \right)\right]\text{.}$$

The functionals $H_{4}$ and $H_{5}$ are functional analogues of standardised empirical processes, specifically

$$T^{-1}\overset{\left[\tau_{0}T\right]}{\underset{t=\left[\tau T\right]+1}{\sum }}{\left(-{\left[\left(1-\tau \right)T\right]}^{-1}\overset{T}{\underset{t=\left[\tau T\right]+1}{\sum }}T_{1}^{-1/2}\sigma^{-1}\mu_{t}\right)}^{2}=\int_{\tau }^{\tau_{0}}{\left(-{\left(1-\tau \right)}^{-1}\int_{\tau_{0}}^{1}X_{T}\left(s_{1}\right)ds\right)}^{2}dr+o_{p,\tau }\left(1\right)=H_{4}\left[X_{T,}\left[\tau T\right]/T\right]+o_{p,\tau }\left(1\right)$$

and

$$T^{-1}\overset{T}{\underset{t=\left[\tau_{0}T\right]+1}{\sum }}{\left(T_{1}^{-1/2}\sigma^{-1}\mu_{t}-{\left[\left(1-\tau \right)T\right]}^{-1}\overset{T}{\underset{t=\left[\tau T\right]+1}{\sum }}T_{1}^{-1/2}\sigma^{-1}\mu_{t}\right)}^{2}=\int_{\tau_{0}}^{1}{\left(X_{T}\left(r_{1}\right)-{\left(1-\tau \right)}^{-1}\int_{\tau_{0}}^{1}X_{T}\left(s_{1}\right)ds\right)}^{2}dr+o_{p,\tau }\left(1\right)=H_{5}\left[X_{T},\left[\tau T\right]/T\right]+o_{p,\tau }\left(1\right)\text{.}$$

Now, given the joint convergence of $\left(X_{T},{\overset{ˆ}{\sigma }}_{\varepsilon }^{2},\sigma^{2},\left[\tau T\right]/T\right)\Rightarrow \left(V\left(r_{1}\right),\sigma_{\varepsilon }^{2},\sigma^{2}1\left(\cdot \right),\tau \right),r_{1}=\frac{r-\tau_{0}}{1-\tau_{0}},r\in \left(\tau_{0},1\right]$ and 1(⋅) is the constant function equal to 1 for all $\tau \in \left[\tau_{l},\tau_{u}\right]$, then the weak convergence result of $\sup_{\tau \in \left[\tau_{l},\tau_{u}\right]}T^{-1}J_{BT}\left(\tau \right)$ follows by applying the CMT given the continuity of $g_{2}\left(\cdot \right)$. □

A change in persistence from I(1) to I(0) can be represented through (1) with the stochastic process

(21) $$\mu_{t}=\left\{ \begin{array}{cc} \overset{\left[\tau_{0}T\right]}{\underset{s=1}{\sum }}\varepsilon_{s} & t=1,\ldots ,\left[\tau_{0}T\right]\\ \overset{\left[\tau_{0}T\right]}{\underset{s=1}{\sum }}\varepsilon_{s}+\varepsilon_{t} & t=\left[\tau_{0}T\right]+1,\ldots ,T \end{array}\right.$$

where the specification of (21) avoids a discontinuity in level at the change point, which would apply if $\sum_{s=1}^{\left[\tau_{0}T\right]}\varepsilon_{s}$ is omitted for $t>\left[\tau_{0}T\right]$. For this case, BT and KBA propose replacing (3) and (4) by the minima of the corresponding expressions, with the following lemma and corollary providing analytical results for these change point estimators. The proof is omitted since it follows the same lines as for Lemma 1, while the corollary again follows immediately from (5).

Lemma 2 *Suppose that* Assumption 1 *holds and* $\tau_{0}\in \left[\tau_{l},\tau_{u}\right]\subset \left(0,1\right)$ *in the model for a change from* I(1) *to* I(0) *given by* (1) and (21)*. Then for given* $\tau \in \left[\tau_{l},\tau_{u}\right]$

$$J_{BT}\left(\tau \right)\Rightarrow {\left(\frac{\tau }{1-\tau }\right)}^{2}\left[\frac{\int_{\tau_{1,0}}^{1}{\left[V\left(r\right)\right]}^{2}dr-\frac{\tau_{0}}{1-\tau }{\left(\int_{\tau_{1,0}}^{1}V\left(r\right)dr\right)}^{2}}{\int_{0}^{\tau_{1,0}}{\left[V\left(r\right)\right]}^{2}dr-\frac{\tau_{0}}{\tau }{\left(\int_{0}^{\tau_{1,0}}V\left(r\right)dr\right)}^{2}}\right],\tau_{1,0}=\tau /\tau_{0},\;\tau <\tau_{0}$$

$$T\;J_{BT}\left(\tau \right)\Rightarrow \frac{1}{1-\tau }{\left(\frac{\tau }{\tau_{0}}\right)}^{2}\frac{\sigma_{\varepsilon }^{2}}{\sigma^{2}}{\left[\int_{0}^{1}{\left[V\left(r\right)\right]}^{2}dr-\frac{\tau_{0}}{\tau }{\left(\int_{0}^{1}V\left(r\right)dr\right)}^{2}\right]}^{-1}\text{,}\;\tau \geq \tau_{0}\text{.}$$

*Consequently,* τ^BT *defined by minimising* $J_{BT}\left(\tau \right)$ *in* (3) *is inconsistent since it converges to a random variable, having asymptotic lower bound of* $\tau_{0}$*.*

Corollary 2 *Suppose that* $\tau_{0}\in \left[\tau_{l},\tau_{u}\right]\subset \left(0,1\right)$ *in the model for a change from* I(1) *to* I(0) *given by* (1) and (21)*. For given* $\tau \in \left[\tau_{l},\tau_{u}\right]$*then*

$$T\;J_{KBA}\left(\tau \right)\Rightarrow \frac{\tau }{{\left(1-\tau \right)}^{2}}\left[\frac{\int_{\tau_{1,0}}^{1}{\left[V\left(r\right)\right]}^{2}dr-\frac{\tau_{0}}{1-\tau }{\left(\int_{\tau_{1,0}}^{1}V\left(r\right)dr\right)}^{2}}{\int_{0}^{\tau_{1,0}}{\left[V\left(r\right)\right]}^{2}dr-\frac{\tau_{0}}{\tau }{\left(\int_{0}^{\tau_{1,0}}V\left(r\right)dr\right)}^{2}}\right],\tau_{1,0}=\tau /\tau_{0},\;\tau <\tau_{0}$$

$$T^{2}J_{KBA}\left(\tau \right)\Rightarrow \left(\frac{\tau }{1-\tau }\right)\left(\frac{1}{\tau_{0}^{2}}\right)\frac{\sigma_{\varepsilon }^{2}}{\sigma^{2}}{\left[\int_{0}^{1}{\left[V\left(r\right)\right]}^{2}dr-\frac{\tau_{0}}{\tau }{\left(\int_{0}^{1}V\left(r\right)dr\right)}^{2}\right]}^{-1}\text{,}\;\tau \geq \tau_{0}\text{.}$$

*Consequently,* τ^KBA *defined by minimising* $J_{KBA}\left(\tau \right)$ *in* (4) *is inconsistent, being a random variable with asymptotic lower bound of* $\tau_{0}$*.*

## Footnotes

1 Generalisation to include other deterministic terms is possible, but the case of a mean is sufficient to illustrate the consequences of allowing for deterministic effects in this context.

2 The term in square brackets in (6) and (8) can also be written as

$$\int_{0}^{1}{\left[\bar{V}\left(r\right)\right]}^{2}dr+\left(\frac{\tau_{0}-\tau }{1-\tau }\right){\left(\int_{0}^{1}V\left(r\right)dr\right)}^{2}\text{,}$$

where $\bar{V}\left(r\right)=V\left(r\right)-\int_{0}^{1}V\left(s\right)ds$. The second term here arises because when $\tau <\tau_{0},{\bar{y}}_{1}$ is computed over [(1−τ)T] observations, rather than the $\left[\left(1-\tau_{0}\right)T\right]$ observations that are truly I(1). For a given Brownian motion process, this term is a monotonically decreasing function of τ.

3 We thank a referee for pointing this out to us.

4 Results for the I(1) to I(0) case can be obtained from the authors on request.

5 Although the number of such replications is not reported, approximately 94% to 98% of these reject the null hypothesis when T=100. For larger sample sizes, the null hypothesis of unchanged order is always rejected.

6 Following Remark 6, the results in Table 2 are computed with the search for the break point taken in steps of 0.01 over the interval τ∈[0.2,0.8], ensuring that the approximations to the asymptotic distributions of Section 2 should improve as T increases.
